# Evidence of Chikungunya virus seroprevalence in Myanmar among dengue-suspected patients and healthy volunteers in 2013, 2015, and 2018

**DOI:** 10.1371/journal.pntd.0009961

**Published:** 2021-12-01

**Authors:** Elizabeth Ajema Chebichi Luvai, Aung Kyaw Kyaw, Nundu Sabiti Sabin, Fuxun Yu, Saw Wut Hmone, Kyaw Zin Thant, Shingo Inoue, Kouichi Morita, Mya Myat Ngwe Tun

**Affiliations:** 1 Department of Virology, Institute of Tropical Medicine (NEKKEN), Nagasaki University, Nagasaki, Japan; 2 Program for Nurturing Global Leaders in Tropical and Emerging Communicable Diseases, Graduate School of Biomedical Sciences, Nagasaki University, Nagasaki, Japan; 3 Department of Biomedical Sciences and Technology, School of Health and Biomedical Sciences, The Technical University of Kenya, Nairobi, Kenya; 4 Department of Medical Research, Ministry of Health and Sports, Yangon, Myanmar; 5 Guizhou Provincial People’s Hospital, Guiyang City, Guizhou Province, China; 6 Department of Pathology, University of Medicine-1, Lanmadaw township, Yangon, Myanmar; Universite de Montreal, CANADA

## Abstract

**Introduction:**

Chikungunya virus (CHIKV) is a mosquito-borne virus known to cause acute febrile illness associated with debilitating polyarthritis. In 2019, several institutions in Myanmar reported a CHIKV outbreak. There are no official reports of CHIKV cases between 2011 and 2018. Therefore, this study sought to determine the seroprevalence of CHIKV infection before the 2019 outbreak.

**Methods:**

A total of 1,544 serum samples were collected from healthy volunteers and patients with febrile illnesses in Yangon, Mandalay, and the Myeik district in 2013, 2015, and 2018. Participants ranged from one month to 65 years of age. Antibody screening was performed with in-house anti-CHIKV IgG and IgM ELISA. A neutralization assay was used as a confirmatory test.

**Results:**

The seroprevalence of anti-CHIKV IgM and anti-CHIKV IgG was 8.9% and 28.6%, respectively, with an overall seropositivity rate of 34.5%. A focus reduction neutralization assay confirmed 32.5% seroprevalence of CHIKV in the study population. Age, health status, and region were significantly associated with neutralizing antibodies (NAbs) and CHIKV seropositivity (*p* < 0.05), while gender was not (*p* = 0.9). Seroprevalence in 2013, 2015, and 2018 was 32.1%, 28.8%, and 37.3%, respectively. Of the clinical symptoms observed in participants with fevers, arthralgia was mainly noted in CHIKV-seropositive patients.

**Conclusion:**

The findings in this study reveal the circulation of CHIKV in Myanmar’s Mandalay, Yangon, and Myeik regions before the 2019 CHIKV outbreak. As no treatment or vaccine for CHIKV exists, the virus must be monitored through systematic surveillance in Myanmar.

## Author summary

Few CHIKV outbreaks have been detected in Myanmar since the first documented case in 1973. After an outbreak, the virus seems to disappear from the region gradually for a few years to more than a decade. In 2019, a CHIKV outbreak was reported in blood donors and children with febrile illness in the Mandalay region. The last official report of a CHIKV outbreak before this was in 2010. Our findings showed evidence of both IgG (28.6%) and IgM (8.9%) antibody circulation against CHIKV. In 2018, the highest seroprevalence rate (37.3%) was found, a probable predictor of the CHIKV outbreak reported in 2019. Additionally, we observed an overall prevalence of 32.5% of circulating anti-CHIKV neutralizing antibodies in the study population. Neutralizing antibodies were observed in patients with febrile illness and healthy volunteers. These findings indicate a continued risk for future outbreaks, reinforcing the need to monitor potential outbreaks in the country.

## Introduction

Chikungunya virus (CHIKV) is an alphavirus in the *Togaviridae* family [1]. The virus is classified as an arthropod-borne virus (arbovirus) transmitted primarily by *Aedes aegypti* and *A*. *albopictus* mosquitoes, which are endemic in tropical and subtropical regions [2–4]. The clinical presentation of CHIKV disease varies from self-limiting undifferentiated febrile illness to debilitating polyarthritis and encephalitis and, in some cases, death may occur [5, 6]. According to the World Health Organization (WHO), CHIKV is an emerging public health threat worldwide [7].

CHIKV was first documented in 1952 in Tanzania [8]. A major outbreak reported in Kenya in 2004 [9] led to the spread of CHIKV to the islands of the Indian Ocean, India, and Southeast Asia, with millions of reported cases [10]. CHIKV arrived in the Americas in 2013 and spread from the Caribbean islands to Brazil in 2014 [11, 12]. Imported cases have been documented in various countries in Europe, North America, East Asia, and the Middle East [1, 13–16] and autochthonous cases have been reported in France and Italy [17, 18].

In Myanmar, the first case of CHIKV occurred in 1973 [19], with subsequent cases reported in 1998, 2006, 2008, and 2010 [20–22]. In 2019, a newspaper stated that the Department of Public Health in Myanmar had identified an outbreak of CHIKV in Kachin State, Nay Pyi Taw, and the Tanintharyi region [23]. In the same year, the GeoSentinel Surveillance Network reported 18 cases of CHIKV infection in travelers returning from Myanmar [15]. Additionally, in 2019, an outbreak of CHIKV infection was detected in 20.5% of children with acute febrile illness and 3.2% of blood donors in the Mandalay region [24].

According to reports, after a large-scale CHIKV outbreak in Myanmar, the virus seems to disappear from the region gradually for a few years to more than a decade [20, 23, 25]. However, countries neighboring Myanmar, such as India, frequently report consistent infection rates of CHIKV [20]. It is unclear what factors trigger multiple institutions’ detection of the virus in the regions where outbreaks occur. CHIKV is transmitted by similar vectors as dengue virus (DENV) and, during the early stages of infection, their primary symptoms are indistinguishable [26–29]. DENV and CHIKV coinfection has been reported in Asia, Africa, and some parts of the Americas [28, 30–33]. The co-circulation and clinical similarities of these arboviruses, as well as the limited capacity for CHIKV testing, have contributed to the underdiagnosis of CHIKV in the regions of Myanmar where the two viruses co-circulate [34, 35].

In Myanmar, there is a lack of active screening and surveillance for CHIKV infection. There were no official reports of CHIKV cases between 2010 and 2019 [15]. Therefore, this study aimed to determine the seroprevalence of CHIKV in healthy volunteers and patients suspected to have dengue fever in 2013, 2015, and 2018.

## Methods

### Ethics statement

Ethical approvals for this study were obtained from the Institutional Ethical Committee on Medical Research Involving Human Subjects in Myanmar (1/2012, 6/2015, 097/2017, and 082/2018) and the Institute of Tropical Medicine Ethical Committee, Nagasaki University, Japan (171207186–2, 191003223, and 200619241). Before sample collection, written informed consent was obtained from both patients and healthy donors and the parents or legal guardians of participating children.

### Samples and study population

This study retrospectively analyzed serum samples that had been used in previous research studies [36–38]. The study population comprised 1,544 healthy volunteers and febrile patients with suspected DENV infection. The study participants were from three distinct regions: Mandalay, Yangon, and Myeik (**S1 Fig**). Mandalay is in the upper Myanmar zone and borders India, China, and Bangladesh. Yangon is located in southern Myanmar and is the largest city in the country. Myeik is a coastal region, part of the Mergui archipelago in the extreme south of Myanmar that borders Thailand.

The healthy volunteers consisted of 934 observably healthy and asymptomatic individuals with no history of hospitalization for at least six months before sample collection. Of the 934 individuals, 421 were selected from three monastic schools in Mandalay, and 513 were selected from private clinics in Yangon during routine medical examinations. All of the samples from healthy volunteers were collected in 2018.

There were approximately 610 individuals suspected of dengue fever, of whom, 104 were from Myeik and 506 were from Mandalay. The samples from symptomatic people were collected in 2013 and 2015 to screen for DENV infection during outbreaks.

### Viruses and cell lines

The CHIKV strain S-27, African prototype, was used for CHIKV IgG indirect ELISA, CHIKV IgM capture ELISA, and neutralization testing. The virus was propagated in C636 mosquito cells and used for viral titration. Vero cells (African green monkey kidney epithelial cells, ATCC CCL-81) were used for neutralization tests.

### Detection of anti-CHIKV IgG

The detection of anti-CHIKV IgG was performed to determine progressive or past CHIKV infection in the populations. To screen for anti-CHIKV IgG in the serum samples, an in-house indirect IgG ELISA was performed using purified CHIKV as the assay antigen [39]. Detection of IgG antibodies was carried out following the procedure described in previous studies [21, 22, 24, 40] with minor adjustments. Briefly, 96-well microplates (Nalge Nunc International, Denmark) were coated with antigen (125 ng/100 uL per well) and diluted in coating buffer, except for the blank wells. The plate was incubated overnight at 4°C. The test samples and positive and negative controls were diluted in 1:1000 phosphate-buffered saline in Tween 20 (PBS-T) in 10% Block Ace (Yukijirushi, Japan) and were distributed into duplicate wells. Subsequently, 1:25,000 diluted horseradish peroxidase (HRP) conjugated anti-human IgG (American Qualex, USA) in PBS-T with 10% Block Ace was added. The color was developed by adding *o*-phenylenediamine dihydrochloride solution (OPD; Sigma Chemical Co, USA) to each well with 0.03% hydrogen peroxide in 0.05 M citrate phosphate buffer (pH 5.0). After incubation for an hour at room temperature, the reaction was stopped with 1N sulfuric acid (1N H_2_SO4), and the optical density was read at 492 nm with Multiscan JX. The IgG titers of patients’ serum samples were determined from the positive standard curve. A sample titer of ≥ 3000 was considered IgG positive. To validate the in-house IgG indirect ELISA, 300 samples were screened, and a 50% focus reduction neutralization assay (FRNT_50_) was utilized as the standard.

### Detection of IgM antibodies

Tests for anti-CHIKV IgM were performed to identify recent CHIKV infections in the population. The presence of anti-CHIKV IgM was detected with an in-house IgM capture ELISA system [21, 22, 24, 40]. All wells, except the blank wells, were coated with anti-human IgM goat IgG (Cappel ICN Pharmaceuticals, USA) in 0.05 M carbonate-bicarbonate buffer (pH 9.6) containing 0.02% sodium azide as the diluent. After overnight incubation and blocking, the test samples and positive and negative controls diluted in PBS-T with 10% Block Ace were distributed into duplicate wells. Subsequently, 128 ELISA units of CHIKV assay antigen (strain S-27, African prototype) were added to each well and incubated for 1 hour at 37°C. A dilution of 1:400 of HRP conjugated anti-CHIKV mouse-derived recombinant E1 monoclonal antibody was added and incubated for 1 hour at 37°C. The reaction color was developed and the optical density reading was performed as previously described for IgG detection. A positive control OD_492_/negative control OD_492_ (P/N) ratio ≥ 2.0 was considered positive. To validate the in-house IgM system, we analyzed randomly selected samples with the human anti-CHIKV Abcam IgM ELISA Kit (ab177848) that has been utilized in other studies as the standard [41, 42].

### Neutralizing assay

The neutralizing activity of antibodies from the IgG- and IgM-positive sera was confirmed with a 50% focus reduction neutralization test (FRNT_50_) as described in previous studies [21, 40]. The heat-treated serum samples were mixed with equal volumes of 40 focus-forming units. After incubation at 37°C for 1 hour, the mixture was transferred to duplicate 96-well plates of confluent Vero cell monolayers. After incubation at 37°C for 1.5 hours, the cells were overlaid with 150 μL of 2% FCS MEM containing 1% methylcellulose 4000 (WAKO Pure Chemical Industries, Japan). The plates were then incubated at 37°C with 5% CO_2_ for 36 hours. After fixing the cells, they were blocked and permeabilized as described in previous studies [21]. Viral foci were detected by immunostaining the cells with anti-CHIKV serum from C57BL/6J mice, peroxidase-conjugated anti-mouse IgG (American Qualex, USA), and DAB substrate (WAKO Pure Chemical Industries, Japan). The endpoint serum dilution that provided a ≥ 50% reduction compared with the mean number of the control well was considered the FRNT_50_ titer. Confirmed CHIKV cases were defined as IgG- or IgM-positive with a neutralization titer of ≥ 10.

### Statistical analysis

Data analysis was performed with GraphPad Prism 9.0.1 (GraphPad Software) and Stata Corp (2019) Stata Statistical Software: Release 16 (StataCorp LLC, College Station, TX).

Chi-square tests were used to determine the differences in proportions of risk factors among groups. An initial analysis produced univariate odds ratio (OR) estimates (95% confidence interval) for the potential risk factors using logistic regression, followed by a multivariable logistic regression model to adjust the OR. Additionally, average marginal effects (AMEs) were calculated. Akaike’s information criterion (AIC) and Bayesian information criteria (BIC) were used to select the best model for the study [43–45]. The Kruskal-Wallis H test and Mann-Whitney U test were used to determine the difference in median among groups. The correlation between anti-CHIKV neutralizing antibodies and IgG or IgM seropositivity was determined with Spearman’s correlation coefficient *r*. All test results were considered statistically significant at *p* < 0.05.

## Results

### Demographic characteristics

The demographic characteristics of the study participants are summarized in **Table 1. T**he study population comprised a total of 1,544 participants, 39.0% (602/1,544) female and 61.0% (942/1,544) male. The median age was 12 and the interquartile range was 7–23 years old. The participants were divided into four age groups based on the risk of acquiring severe CHIKV disease [46–50]: ≤ 5 years old, 6–15 years old, 16–45 years old, and ≥ 46 years old. Most of the participants (45.4%) were school-aged children (6–15 years old). The Mandalay region had the highest proportion of participants (60.0%), whereas Myeik had the lowest (6.8%). The study participants were also grouped by health status: healthy volunteers represented 60.5% of participants and febrile-illness patients, 39.5%. Most of the study participants (60.5%) were sampled in 2018, followed by 21.4% in 2015 and 18.1% in 2013.

**Table 1:** Demographic characteristics of the study population (N = 1,544).

Variable	Overall number (%)	Female (%)	Male (%)
Age* (years)			
≤5	268 (17.4)	134 (50.0)	134 (50.0)
6–15	701 (45.4)	328 (47.8)	373 (53.2)
16–45	554(35.9)	134 (24.2	420(75.8)
≥46	21 (1.3)	6 (28.6)	15 (71.4)
**Region**			
Yangon	513 (33.2)	127 (24.7)	386 (75.2)
Mandalay	927 (60.0)	425 (42.9)	502 (54.1)
Myeik	104 (6.8)	50 (48.0)	54 (51.9)
**Health status**			
Febrile patients	610 (39.5)	302 (49.5)	308 (50.5)
Healthy volunteers	934 (60.5)	300 (32.1)	634 (67.9)
**Year of collection**			
2013	280 (18.1)	133 (47.5)	147 (52.5)
2015	330 (21.4)	169 (51.2)	161 (48.8)
2018	934 (60.5)	300 (32.1)	634 (67.9)

* The participants’ ages were defined based on the date and year of sample collection.

### CHIKV IgG and IgM seroprevalence in the study population

The validation results showed that the sensitivity and specificity of the in-house anti-CHIKV IgM capture ELISA were 98.3% and 88%, respectively (**S1 Table**). Additionally, the sensitivity and specificity of the anti-CHIKV IgG indirect ELISA were 94.2% and 100%, respectively (**S2 Table**).

Of the 1,544 serum samples tested, 28.6% were positive for anti-CHIKV IgG and 8.9% were positive for anti-CHIKV IgM (**Table 2**). Overall, the seroprevalence of CHIKV IgG and IgM antibodies was 34.5% (**Table 2**). Approximately 3.0% of the study population had both IgG and IgM antibodies. Despite representing the smallest group of participants in the study, the older population (≥ 46 years) had the highest seroprevalence (85.7%), followed by the 16–45-year-old group (44.4%). The 6–15-year-old group and the children ≤ 5 years old had seroprevalences of 29.2% and 23.9%, respectively. Participants from Yangon exhibited the highest seroprevalence rate (47.9%), followed by those from Myeik (42.3%), and those from Mandalay had the lowest seroprevalence at 26.2%. The seroprevalence rate was higher in males (36.2%) compared with females (31.9%). Notably, the overall seroprevalence rate was lower in the febrile-patient population (30.3%) than among the healthy volunteers (37.3%). Samples taken in 2018 showed the highest seropositivity (37.3%), followed by those from 2013 (32.1%) and 2015 (28.8%) (**Table 2**).

**Table 2:** Anti-CHIKV seropositivity rate in the study population (N = 1,544).

Variable	Number	IgG positive (%)	IgM positive (%)	IgG and IgM positive (%)	IgG and/or IgM positive (%)
**Age (years)**					
≤5	268	40 (14.9)	27 (10.1)	3 (7.5)	64 (23.9)
6–15	701	164 (23.4)	61 (8.7)	20 (12.2)	205 (29.2)
16–45	554	221 (39.9)	46 (8.3)	21 (9.5)	246 (44.4)
≥46	21	17 (80.9)	3 (14.3)	2 (11.8)	18 (85.7)
**Region**					
Mandalay	927	187 (20.2)	75 (8.1)	19 (10.2)	243 (26.2)
Myeik	104	33 (31.7)	19 (18.3)	8 (24.2	44 (42.3)
Yangon	513	222 (43.6)	43 (8.4)	19 (8.6)	246 (47.9)
**Gender**					
Female	602	154 (25.6)	51 (8.5)	13 (8.4)	192 (31.9)
Male	942	288 (30.5)	86 (9.2)	33 (11.5)	341 (36.2)
**Health status**					
Febrile patients	610	152 (24.9)	48 (7.9)	15 (9.7)	185 (30.3)
Healthy volunteers	934	290 (31.1)	89 (9.5)	31 (10.7)	348 (37.3)
**Year**					
**2013**	280	83 (29.6)	13 (4.6)	6 (7.2)	90 (32.1)
**2015**	330	69 (20.9)	35 (10.6)	9 (13.0)	95 (28.8)
**2018**	934	290 (31.1)	89 (9.5)	31 (10.7)	348 (37.3)
**Overall**	1544	442 (28.6)	137 (8.9)	46 (3.0)	533 (34.5)

**Fig 1:**
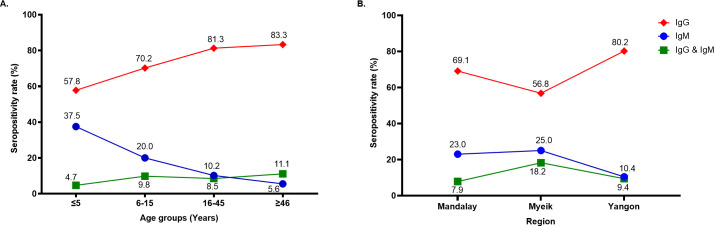
CHIKV IgG and IgM distribution among the seropositive population.

Further analysis to determine the distribution of CHIKV antibodies among the seropositive population revealed that the amount of anti-CHIKV IgG increased with increasing age (**Fig 1A**). In contrast, anti-CHIKV IgM seroprevalence increased with decreasing age, with school-aged children (6–15 years) having the highest seroprevalence rate (10.2%) of individuals with both IgM and IgG CHIKV antibodies (**Fig 1A**). A high anti-CHIKV IgG seroprevalence rate was observed in samples taken from Mandalay (69.1%) and Yangon (80.2%), whereas Myeik had the highest distribution of anti-CHIKV IgM antibodies (25.0%) (**Fig 1B**).

The percentage of anti-CHIKV IgG and IgM and the presence of both IgG and IgM among the seropositive population by age group (A) and region (B) are indicated in the graphs.

The association of the independent variables (age, region, gender, and health status) with the IgM or IgG seropositivity rate is illustrated in **Table 3**. In the multivariable logistic regression model, age, region, and health status were significantly associated with the seroprevalence rate (*p* < 0.05) **Table 3**. Using the multivariable logistic regression model, we found that children ≥ 6 years old were 1.7 times (95% CI: 1.2–2.4) more likely to be CHIKV-seropositive than children ≤ 5 years old, and individuals 16–45 years old were 1.9 times (95% CI: 1.0–3.8) more likely to be CHIKV-seropositive than children ≤ 5 years old. Individuals ≥ 46 years old were 13.4 times (95% CI: 3.3–54.7) more likely to be CHIKV-seropositive compared to children ≤ 5 years old. The adjusted AMEs estimates of CHIKV seropositivity among the 6–15-year-old, 16–45-year-old, and ≥ 46-year-old age groups were 0.11% (95% CI: 0.04%–0.17%), 0.11% (95% CI: -0.02%–0.2%) and 0.53% (0.3%–0.8%), respectively, higher than children ≤ 5 years (**S5 Table**). Furthermore, individuals from Myeik were 1.9 times (95% CI: 1.2–3.0), and Yangon, 2.4 times (95% CI: 1.3–4.2) more likely to be CHIKV-seropositive compared with those from Mandalay (**Table 3**). Additionally, the adjusted AMEs estimates of CHIKV seropositivity were 0.14% (95% CI: 0.04%–0.2%) and 0.19% (95% CI: 0.06%–0.3%) higher in Myeik and Yangon, respectively, than in Mandalay (**S5 Table**). Notably, the febrile patients were 1.5 times (95% CI: 1.1–2.1) more likely to be CHIKV-seropositive than the healthy volunteers. The adjusted AMEs estimate of CHIKV seroprevalence among the febrile patients was 0.09% (95% CI: 0.02%–0.15%) higher than in healthy volunteers.

**Table 3:** Association of CHIKV seroprevalence rate with age, region, gender, and health status (N = 1,544).

			Univariable analysis		Multivariable analysis	
Variable	Number	IgG and/or IgM positive (%)	cOR* (95% CI)	*p*-value	aOR* (95% CI)	*p*-value
**Age (years)**						
≤5	268	23.9	Ref.		Ref.	
6–15	701	29.2	1.3 (0.9–1.8)	0.096	1.7 (1.2–2.4)	**0.004**
16–45	554	44.4	2.5 (1.8–3.5)	**<0.0001**	1.9 (1.0–3.8)	**0.05**
≥46	21	85.7	19.1 (5.5–67.0)	**<0.0001**	13.4 (3.3–54.7)	**<0.0001**
**Region**						
Mandalay	927	26.2	Ref.		Ref.	
Myeik	104	42.3	2.1 (1.4–3.1)	**0.001**	1.9 (1.2–3.0)	**0.003**
Yangon	513	47.9	2.6 (2.1–3.3)	**<0.001**	2.4 (1.3–4.2)	**0.004**
**Gender**						
Female	602	31.9	Ref.		Ref.	
Male	942	36.2	1.2 (1.0–1.5)	0.083	1.0 (0.8–1.3)	0.8
**Health status**						
Healthy volunteers	934	37.3	Ref.		Ref.	
Febrile patients	610	30.3	1.4 (1.1–1.6)	**0.005**	1.5 (1.1–2.1)	**0.02**

CHIKV: Chikungunya virus; cOR: crude odds ratio; aOR: adjusted odds ratio; CI: confidence interval. Seroprevalence rate includes both IgG and IgM positive results.

### Seroprevalence of anti-CHIKV IgG and IgM in 2013, 2015, and 2018

**Fig 2:**
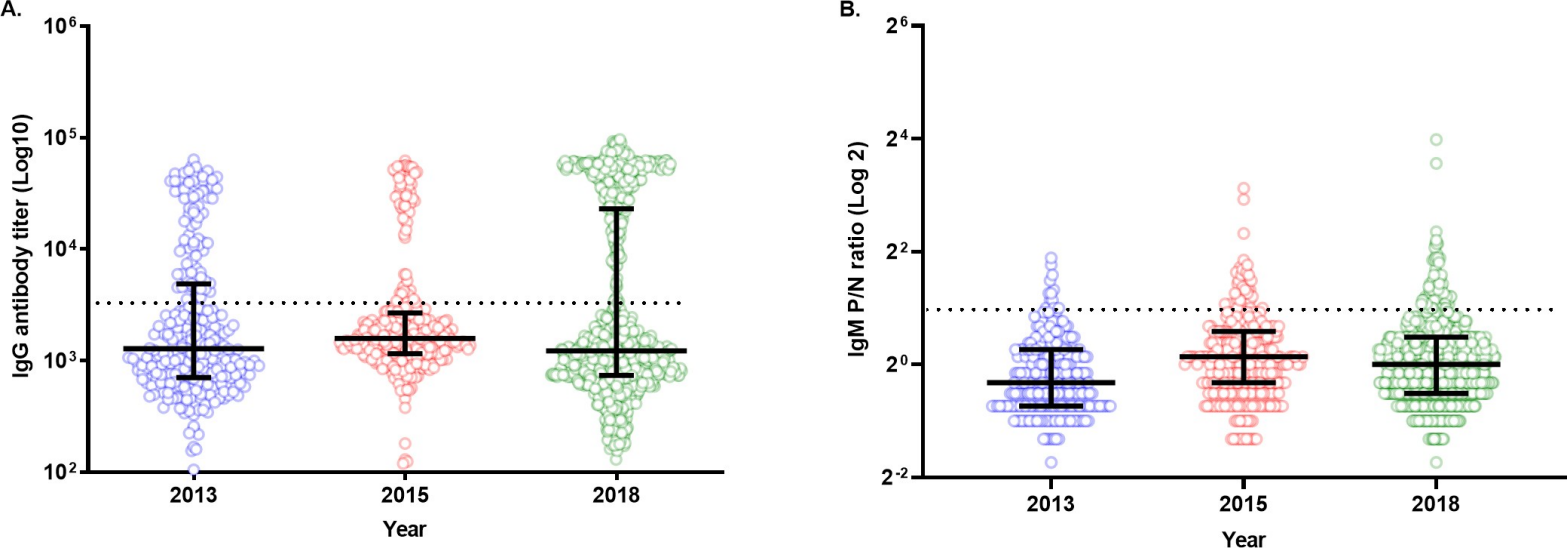
Distribution of anti-CHIKV IgG and IgM antibodies in 2013, 2015, and 2018. (A) An anti-CHIKV IgG titer of ≥ 3000 was considered CHIKV-positive and is indicated by a black dotted line. (B) An anti-CHIKV IgM-positive/negative (P/N) ratio of ≥ 2.0 was considered IgM-positive and is denoted by a black dotted line. Each dot represents the sample distribution of IgG and IgM in the study population, and the black error bars denote the median and interquartile ranges in figures A and B, respectively.

The highest distribution of anti-CHIKV IgG antibodies was observed in the 2018 group (290/934), followed by 2013 (83/280) and 2015 (69/330), indicating a lower circulation of the antibodies in earlier years (**Fig 2A**). Samples from 2015 showed a high circulation of IgG with a lower titer, as indicated by the upper quartile range below the cutoff line (**Fig 2A**). Samples from 2013 had the lowest distribution of IgM antibodies (13/280), whereas those from 2015 (35/330) and 2018 (89/934) showed a fairly higher circulation of the antibodies in the study population (**Fig 2B**).

### Prevalence of anti-CHIKV neutralizing antibodies (NAbs) in the study population

**Fig 3:**
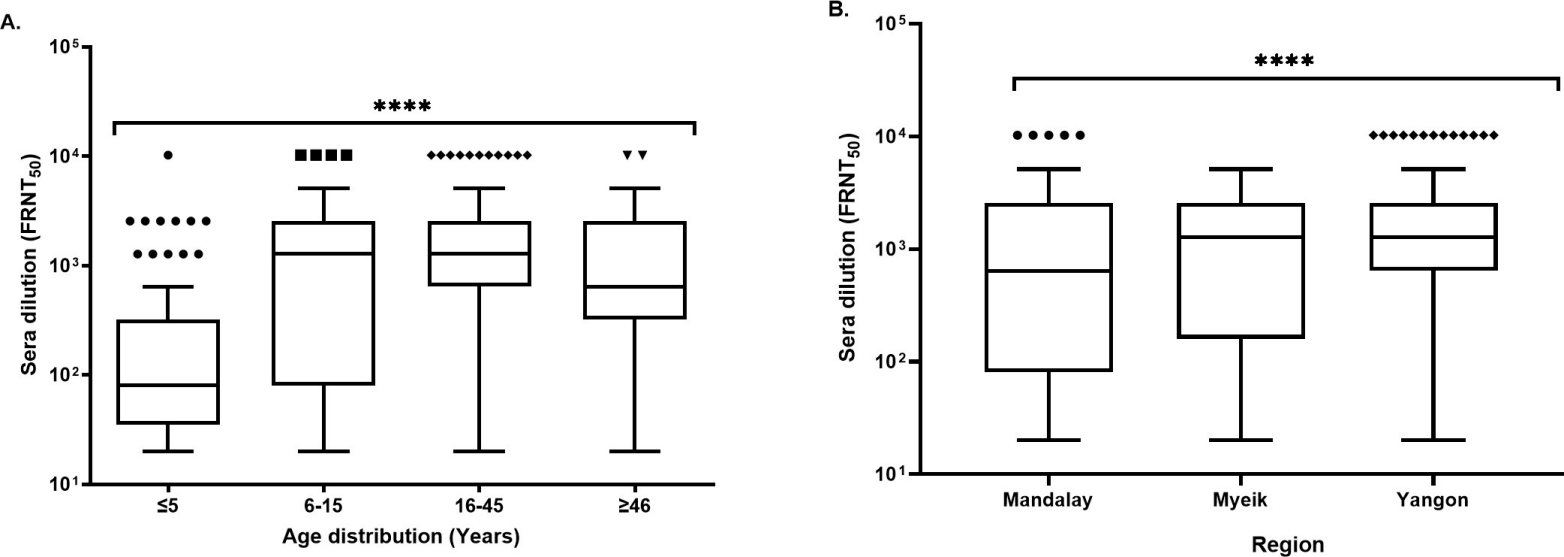
Distribution of NAbs within the study population. The presence of anti-CHIKV NAbs in the serum of participants was categorized according to age group (A) and region (B). Each dot above the whiskers indicates the outliers observed in each group. A Kruskal-Wallis H test was used to determine statistical significance among the age groups and regions, *p* < 0.0001 is denoted by ****.

**Fig 4:**
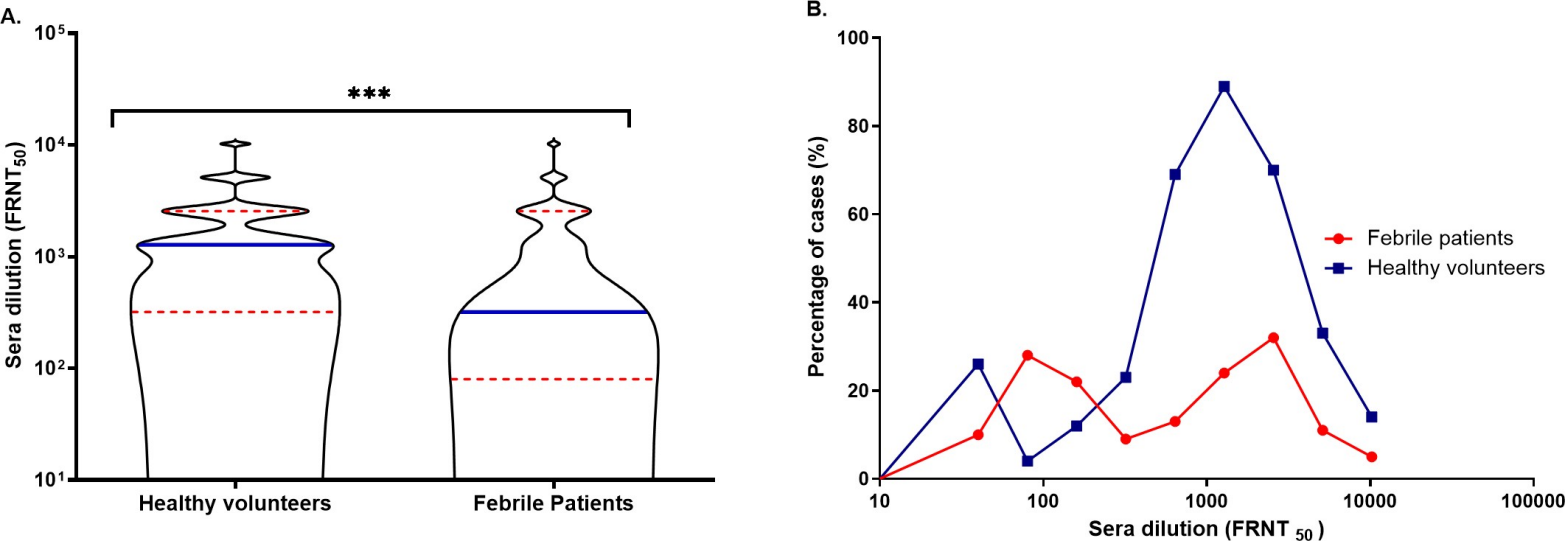
Distribution of CHIKV NAbs among healthy volunteers and febrile patients. A) Distribution of NAbs was computed as a continuous approximation of the probability density using Kernel density estimation (KDE). The densities of both populations are annotated with the median (blue straight line) and the interquartile range (red dotted lines). The distribution of healthy volunteers’ NAbs shows the median of samples above the 10^3^ titer. The probability density of febrile patients is concentrated below the 10^3^ NAb titer. Statistical significance was measured with the Mann-Whitney U test, *p* = 0.007 is denoted by ***. B) Comparison of the proportion of the NAb titer in healthy volunteers (blue) and febrile patients (red).

All the IgM and IgG seropositive samples were confirmed CHIKV-positive by 50% focus reduction neutralizing titer (FRNT_50_). Overall, the anti-CHIKV neutralizing antibody prevalence rate was confirmed at 32.5% (502/1,544). The FRNT_50_ titers ranged from 20–10,240. The older age group (≥ 46 years) had the highest mean NAb titer of 1,885 (95% CI: 498–3,272), however, the geometric mean antibody titer (GMT) was 806 (95% CI: 352–1,845) (**S7 Table**). The Yangon region had the highest mean NAb titer, 923 (95% CI: 756–1,091). The geometric mean antibody titer values (GMT) patterns were more or less similar to the mean NAb titers observed for the region, gender, health status, and year (**S7 Table**). The median NAb titers for the ≤ 5 years old age group were the lowest compared with the other three age groups **(Fig 3A**), which is consistent with the pattern of the mean NAb titers (**S7 Table**). The adult age group (16–45 years) had a majority of samples with a high NAb titer, with several samples above the 10^4^ range **(Fig 3A**). The differences among each age group were statistically significant (*p* < 0.0001). Most high NAb titers were from the Yangon region, whereas Mandalay had NAbs with both the lowest and highest titers (**Fig 3B**). The NAb interquartile ranges and median titers of healthy volunteers were significantly higher than those in febrile patients (*p* = 0.007) (**Fig 4A**). Additionally, **Fig 4B** shows that a higher percentage of healthy volunteers had titers above 10^3^, whereas most febrile patients had much lower titers.

Further analysis was performed to determine the association between independent factors (age, region, gender, and health status) and CHIKV NAb prevalence (**Table 4**). As in the seropositivity results, in the multivariable logistic regression model, age, region, and health status were significantly associated with NAb prevalence, while gender was not (*p* = 0.9). The multivariable logistic regression model indicated that the odds of CHIKV NAb prevalence were 1.7 times (95% CI: 1.2–2.6) and 2.1 times (95% CI: 1.1–4.2) higher among children ≥ 6 years old and adults 16–45 years old, respectively, than in children ≤ 5 years old (**Table 4**). Interestingly, the odds of CHIKV NAb prevalence were 16.1 times (95% CI: 4.0–65.7) higher in the ≥ 46-year-old group than in children ≤ 5 years old (**Table 4**). The adjusted AMEs estimates of CHIKV NAb prevalence among the 6–15-year-old, 16–45-year-old, and ≥ 46-year-old age groups were 0.11% (95% CI: 0.04%–0.17%), 0.15% (95% CI: 0.02%–0.28%), and 0.59%; (0.36%–0.81%), respectively; higher than in children ≤ 5 years old (**S6 Table**). Notably, the study participants from Yangon were 2.4 times (95% CI: 1.3–4.3), and Myeik, 1.9 times (95% CI: 1.2–2.9), more likely to have high CHIKV NAbs compared with those from Mandalay, as shown in **Table 4**. Additionally, the adjusted AMEs of CHIKV NAbs were 0.13% (95% CI: 0.03%–0.23%) and 0.19% (95% CI: 0.06%–0.32%) higher in Myeik and Yangon, respectively, than in Mandalay (**S6 Table**). As previously observed in the seropositivity results, the prevalence of CHIKV NAb was 1.6 times (95% CI: 1.2–2.2) higher in febrile patients than in healthy volunteers (**Table 4**). The adjusted AMEs estimate of CHIKV NAb prevalence was 0.1% (95% CI: 0.03%–0.16%) higher in febrile patients than in healthy volunteers (**S6 Table**).

**Table 4:** Association of the anti-CHIKV NAbs with age, region, gender, and health status (N = 1,544).

			Univariable analysis		Multivariable analysis	
Variable	Number	NAbs (%)	cOR*(95%CI)	*p-*value	aOR*(95%CI)	*p-*value
**Age (years)**						
≤5	268	21.6	Ref.		Ref.	
6–15	701	27.3	1.4 (1.0–1.9)	0.075	1.7 (1.2–2.6)	**0.002**
16–45	554	42.4	2.6(1.9–3.7)	**<0.0001**	2.1 (1.1–4.2)	**0.03**
**≥46**	21	85.7	21.7 (6.2–76.3)	<0.0001	16.1 (4.0–65.7)	0.0001
**Region**						
Mandalay	927	24.3	Ref.		Ref.	
Myeik	104	39.4	2.0 (1.3–3.1)	**0.001**	1.9 (1.2–2.9)	**0.006**
Yangon	513	46.0	2.7 (2.1–3.3)	**<0.0001**	2.4 (1.3–4.3)	**0.004**
**Gender**						
Female	602	29.9	Ref.		Ref.	
Male	942	34.2	1.2 (1.0–1.5)	**0.08**	1.0 (0.8–1.3)	0.8
**Health status**						
Healthy volunteers	934	35.1	Ref.		Ref.	
Febrile patients	610	28.5	0.7 (0.6–0.9)	**0.007**	1.6 (1.2–2.2)	0.005

NAbs: Neutralizing antibodies; cOR: crude odds ratio; aOR: adjusted odds ratio; CI: confidence interval.

### Correlation between CHIKV IgG and IgM antibodies with NAbs

**Fig 5:**
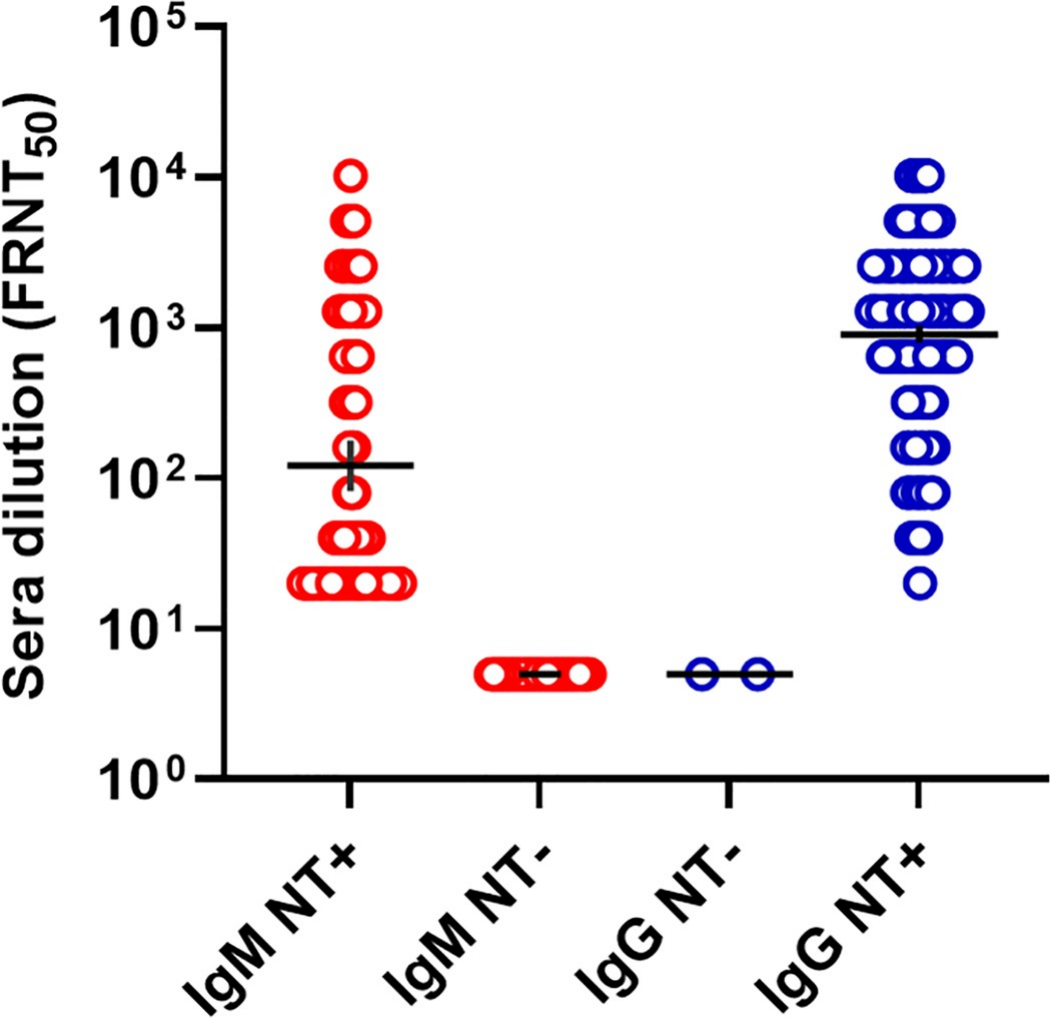
Comparison between CHIKV antibody titer and NAbs.

A higher number of anti-CHIKV IgG antibodies were able to neutralize the virus than anti-CHIKV IgM antibodies (**Fig 5**). The anti-CHIKV IgG titer and NAb titer were positively and significantly correlated (r = 0.8; 95% CI: 0.7–0.8, *p* < 0.0001) as shown in **S2A Fig**. Notably, the correlation between anti-CHIKV IgM titer and NAb titer (r = 0.05; 95% CI: 0.002–0.1, *p* = 0.04) was weaker, as shown in **S2B Fig**. Anti-CHIKV IgG antibodies had a higher NAb titer than IgM antibodies, illustrated by the geometric mean titer (with 95% CI bars) given in **Fig 5**. Approximately 440/442 anti-CHIKV IgG antibodies were able to neutralize the virus, compared with 105/137 of the anti-CHIKV IgM antibodies (**Fig 5**).

Sero-neutralization was performed on sera diluted at 1:10 to detect neutralizing IgM and IgG. The presence and absence of neutralizing titers (NT) of IgM and IgG were denoted by NT+ and NT-, respectively. The black bars indicate the geometric mean titers and 95% CI for both IgG and IgM.

### Presence of anti-CHIKV and anti-DENV antibodies in the febrile patient population

To further compare CHIKV infection in febrile patients in this study population, data from previous studies were utilized to evaluate the presence of DENV in the same population [36, 37]. The flow chart (**S3 Fig**) shows that, of the 610 patients, 30.3% were CHIKV-seropositive, and 5.9% of these had only CHIKV antibodies while 24.4% had both DENV and CHIKV antibodies. Upon evaluation of the specific signs and symptoms that were observed in the febrile patient population, arthralgia was observed in CHIKV cases and dual DENV- and CHIKV-seropositive individuals (**S4 Fig)**.

## Discussion

CHIKV infection has become a major public health concern globally and poses a significant socio-economic burden. The findings in this paper confirm the circulation of CHIKV in 2013, 2015, and 2018, and one-third of the study participants were confirmed CHIKV-seropositive. We demonstrated the occurrence of CHIKV infection among dengue-suspected patients and healthy volunteers in three distinct regions in Myanmar. This study revealed that 28.6% of the participants had anti-CHIKV IgG antibodies, 8.9% had anti-CHIKV IgM antibodies, and 3.0% had both IgM and IgG anti-CHIKV antibodies, with 34.5% overall seropositivity.

The study revealed the highest seroprevalence rate (37.3%) in 2018, a probable predictor of the outbreak reported in 2019 [15, 23, 24]. Around the same period, neighboring countries, such as Thailand and India, reported CHIKV seroprevalence rates of 26.8% and 18.1%, respectively [51, 52]. Interestingly, our study revealed that 2013 also had a high seroprevalence rate (32.1%), however, IgM seroprevalence was lower compared with the other tested years. High anti-CHIKV IgG seroprevalence in 2013 might be attributed to past infections. In contrast, 2015 showed the highest CHIKV IgM antibody circulation (10.7%), indicating infection within that year. The high seroprevalence rate could be attributed to cross-border spread as neighboring countries, such as Laos and Bangladesh, reported high CHIKV seroprevalence (43–90%) around the same period in which our samples were collected [53, 54].

In 2010, a study reported that the seroprevalence of CHIKV in Mandalay was 5.2% [21]. Our findings show an increase to 26.2% in that region. By using an adjusted odds ratio (aOR), we determined that participants from Myeik (aOR: 1.9, 95% CI: 1.2–3.0) and Yangon (aOR: 2.4, 95% CI: 1.3–4.2) are approximately two times more at risk of CHIKV infection than those from Mandalay. The reason the CHIKV seropositivity rate in Mandalay is lower than the other two cities, despite previous reports of the high endemicity of other arboviruses transmitted by similar vectors, is unclear [36–38, 55]. Both IgG and IgM seropositivity were observed at all the study sites, indicating ongoing infection despite previous exposure, and also suggesting that CHIKV endemicity is maintained within the studied regions [56].

This study’s findings revealed that CHIKV seroprevalence increased with age, from 23.9% among children ≤ 5 years old to 29.2% (aOR: 1.7, 95% CI: 1.2–2.4) among 6–15-year-old children and 44.4% (aOR: 1.9, 95% CI: 1.0–3.8) among adults 16–45 years old. Notably, the older age group (≥ 46 years) had a high aOR of 13.4 (95% CI: 3.3–54.7), indicating that this age group had the highest seroprevalence of CHIKV infection. One of the reported risk factors for acquiring chronic CHIKV disease is being more than 45 years old [48, 57]. A similar trend was observed among age groups in studies conducted in India, Singapore, and Nigeria [52, 56, 58]. The relationship between increasing age and CHIKV susceptibility has been attributed to the increased degree of exposure and impaired immune function in the elderly population [58, 59]. However, the seroprevalence of anti-CHIKV IgM antibodies was highest among children ≤ 5 years old and adults ≥ 46 years old, which is indicative of acute infection in the population. The ≤ 5-year-old and ≥ 46-year-old age groups are at the highest risk of severe disease due to CHIKV infection [46–50]. Infections in young children typically occur either through vertical transmission or through mosquito bites [60]. Interestingly, CHIKV seropositivity was not significantly associated with gender, as indicated by an adjusted OR of 1.0 (95% CI: 0.8–1.3). A study conducted in India reported a similar finding [52], although other studies showed significant differences in seropositivity by gender [56, 58].

The correlation between IgG and neutralizing antibodies (NAbs) (r = 0.76, 95% CI: 0.71–0.79) was significantly higher than that of IgM antibodies (r = 0.05, 95% CI: 0.002–0.1). Our findings agree with previous studies in which anti-CHIKV IgM antibodies have been shown to have weaker neutralizing effects than IgG [61]. Reportedly, ten days after disease onset, IgM plays a minimal role in overall neutralizing activity because neutralizing IgG becomes dominant [62]. This mechanism explains why our findings showed more IgG antibodies able to neutralize the virus and primarily indicated with a high titer.

The overall prevalence of NAbs in the study population was 32.5%. As observed with CHIKV seropositivity, the association of age and region with NAbs was statistically significant. Similarly, the NAb prevalence rate increased with increasing age, as in CHIKV seropositivity. Additionally, by using an adjusted odds ratio, we determined that participants from Myeik (1.9; 95% CI: 1.2–2.9) and Yangon (2.4; 95% CI: 1.3–4.3) were approximately two times more likely to have anti-CHIKV neutralizing antibodies than those from Mandalay. Similarly, no significant difference was observed in the prevalence of NAbs by gender (*p* < 0.05). This finding indicates that a third of the study population is protected from infection, as other studies have shown that the presence of CHIKV NAbs with titers ≥1:10 is correlated with protection from symptomatic infection and subclinical seroconversion [63–65].

The prevalence of CHIKV NAbs was higher in healthy volunteers (35.1%) than patients with febrile illness (28.1%). In contrast, the adjusted odds ratio revealed that CHIKV NAb prevalence was 1.6 times (95% CI: 1.2–2.2) higher in febrile patients than healthy volunteers. Furthermore, the adjusted AMEs estimates revealed that CHIKV NAb prevalence was 0.10% (95% CI: 0.03–0.16) higher in febrile patients than healthy volunteers. The higher proportion of CHIKV NAbs among healthy volunteers compared with febrile patients is attributed to the high number of DENV-seropositive individuals in the population. The samples from the febrile patients had been collected for DENV seroprevalence screening [36, 37]. 30.3% of the febrile patients had CHIKV antibodies, whereas 24.4% had both DENV and CHIKV antibodies. CHIKV and DENV normally co-circulate because they have similar vectors and are endemic in tropical regions [66]. The clinical symptoms observed in DENV- and CHIKV-positive individuals are initially similar; however, some distinguishing features are typically observed [66]. All of the clinical symptoms reported were observed in either CHIKV- or DENV-seropositive cases. However, 33.3% of arthralgia was noted in CHIKV-seropositive patients and 66.6% in combined CHIKV- and DENV-seropositive patients, but not in DENV-only cases. Arthralgia and rash have been identified as some of the typical clinical features of CHIKV although, in some cases, the infection can progress to debilitating polyarthritis that can last for months and years [67].

The findings in this study confirm the circulation of CHIKV in both healthy volunteers and febrile patients. However, this study had several limitations. The samples used in this work were collected previously for various studies, and this is apparent in the differing representation of each region. Additionally, the age groups were not evenly represented in the sampled regions. Lastly, the study was not able to show a systematic trend of infection between 2013 and 2018 because we lacked samples for 2014, 2016, and 2017. A more systematic seroprevalence study should be conducted to determine the extent of CHIKV infection among the population of Myanmar.

This study revealed the circulation of CHIKV in Mandalay, Yangon, and Myeik, which are located in northern Myanmar, southern Myanmar, and the extreme south of the country respectively; this indicates the presence of the virus in the country. The sample population had an overall IgG and IgM seropositivity rate of 34.5% (533/1,544) and a NAb prevalence rate of 32.5% (502/1,544). Additionally, our findings demonstrated the co-circulation of CHIKV with DENV antibodies in patients with febrile illness. This finding strengthens the need to incorporate screening for CHIKV during DENV outbreaks. With no current CHIKV treatment or vaccine, continuous monitoring of the virus through systematic surveillance is necessary. Lastly, the development of an affordable, reliable, and rapid diagnostic tool to detect multiple viruses that co-circulate is necessary for continuous seroprevalence surveillance in the region.

## Supporting information

S1 Fig Map of Myanmar showing the three study sites. The regions marked in black are Mandalay, Yangon, and Myeik. Source: https://aseanup.com/free-maps-myanmar/.
(TIF)

S2 Fig Correlation between anti-CHIKV IgG/IgM antibodies and neutralizing antibodies (NAbs). Spearman’s correlation coefficient *r* was used to determine the relationship between CHIKV IgG/IgM antibodies and NAbs. A) The red dotted horizontal and vertical lines represent the cutoff points for neutralization-positive (≥ 10) and IgG-positive (≥ 3000) samples, respectively. B) The red dotted horizontal and vertical lines represent the cutoff points for neutralization-positive (≥10) and IgM-positive (Positive–negative ratio ≥ 2) samples, respectively. *p* values < 0.05 were considered statistically significant.
(TIF)

S3 Fig Seroprevalence of CHIKV and DENV among febrile patients. The flow chart illustrates the seroprevalence rates of DENV, CHIKV, and DENV–CHIKV infections.
(TIF)

S4 Fig Comparison of clinical symptoms among CHIKV- and DENV-infected patients. The prevalence rate of the clinical presentation among the febrile patients is indicated on each bar.
(TIF)

S1 Table Validation of in-house anti-CHIKV IgM-capture ELISA with human anti-CHIKV Abcam IgM ELISA Kit (ab177848) as the standard. The sensitivity of the in-house anti-CHIKV IgM capture ELISA was 98.3% (95% CI: 90.9%–100%) and specificity was 88.0% (95% CI: 71.8%–96.6%), with an accuracy of 94.6%.
(DOCX)

S2 Table Validation of the in-house anti-CHIKV IgG indirect ELISA with FRNT_50_ as the standard. The sensitivity of the in-house anti-CHIKV IgG indirect ELISA was 94.2% (95% CI: 88.9%–97.5%) and specificity was 100% (97.8%–100%), with an accuracy of 97.4%.
(DOCX)

S3 Table Model selection criteria for the association of CHIKV seropositivity with independent variables. Key: AIC, Akaike’s Information Criterion; BIC, Bayesian Information Criterion. The logistic regression model with four independent variables was selected because it had the lowest AIC and BIC values. The model was correctly classified at 66.4% and the goodness of fit test was *p* = 0.3773.
(DOCX)

S4 Table Model selection criteria for the association of CHIKV neutralizing antibodies with independent variables. Key: AIC, Akaike’s Information Criterion; BIC, Bayesian Information Criterion. The logistic regression model with the four independent variables was selected because it had the lowest AIC and BIC values. The model was correctly classified at 68.5%, and the goodness of fit test was *p* = 0.2289.
(DOCX)

S5 Table Average marginal effects (AMEs) estimates of CHIKV seroprevalence by age, region, gender, and health status. The *p*-values highlighted in bold indicate significant values.
(DOCX)

S6 Table Average marginal effects (AMEs) estimates of CHIKV NAb prevalence by age, region, gender, and health status. The *p*-values highlighted in bold indicate significant values.
(DOCX)

S7 Table Anti-CHIKV neutralizing antibody (NAb) mean titers and geometric mean titers (GMT) categorized by age, region, gender, health status, and year. (DOCX)
