# Supplementary figures and images for: Evidence of Chikungunya virus seroprevalence in Myanmar among dengue-suspected patients and healthy volunteers in 2013, 2015, and 2018
Source: PLoS Negl Trop Dis. 2021 Dec 1;15(12):e0009961. doi: 10.1371/journal.pntd.0009961 (PMC8635363; doi:10.1371/journal.pntd.0009961)

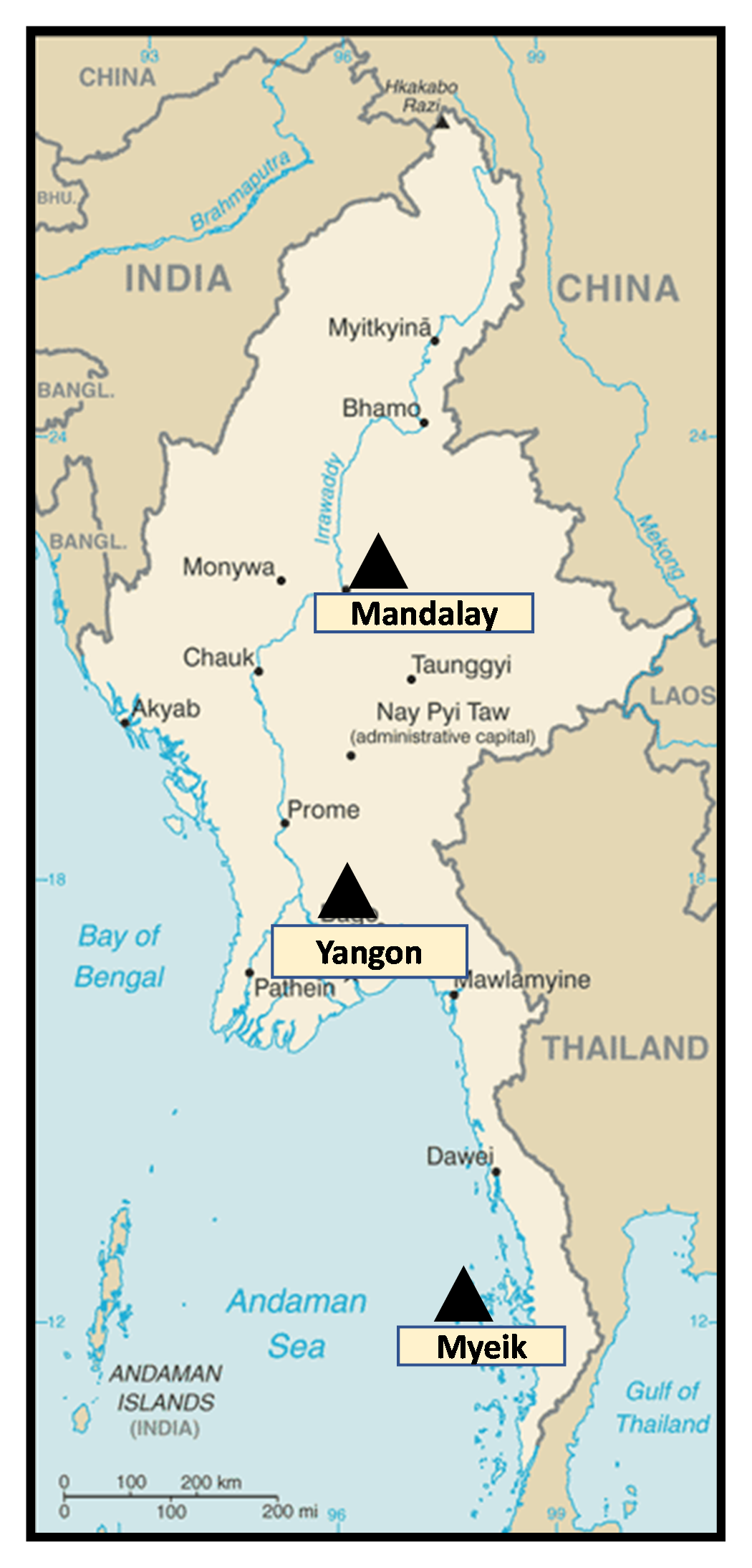

Supplement: S1 Fig — The regions marked in black are Mandalay, Yangon, and Myeik. Source: https://aseanup.com/free-maps-myanmar/. (TIF) [file pntd.0009961.s001.tif]

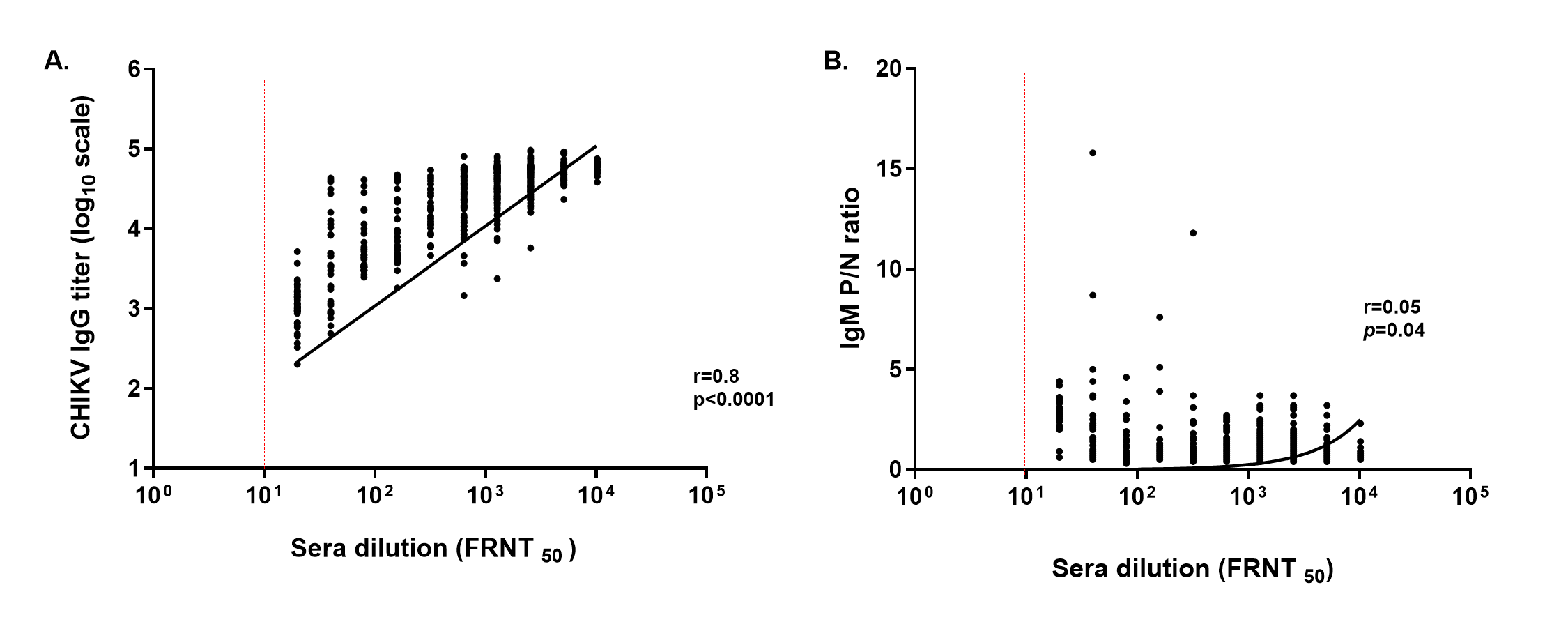

Supplement: S2 Fig — Spearman’s correlation coefficient r was used to determine the relationship between CHIKV IgG/IgM antibodies and NAbs. A) The red dotted horizontal and vertical lines represent the cutoff points for neutralization-positive (≥ 10) and IgG-positive (≥ 3000) samples, respectively. B) The red dotted horizontal and vertical lines represent the cutoff points for neutralization-positive (≥10) and IgM-positive (Positive–negative ratio ≥ 2) samples, respectively. p values < 0.05 were considered statistically significant. (TIF) [file pntd.0009961.s002.tif]

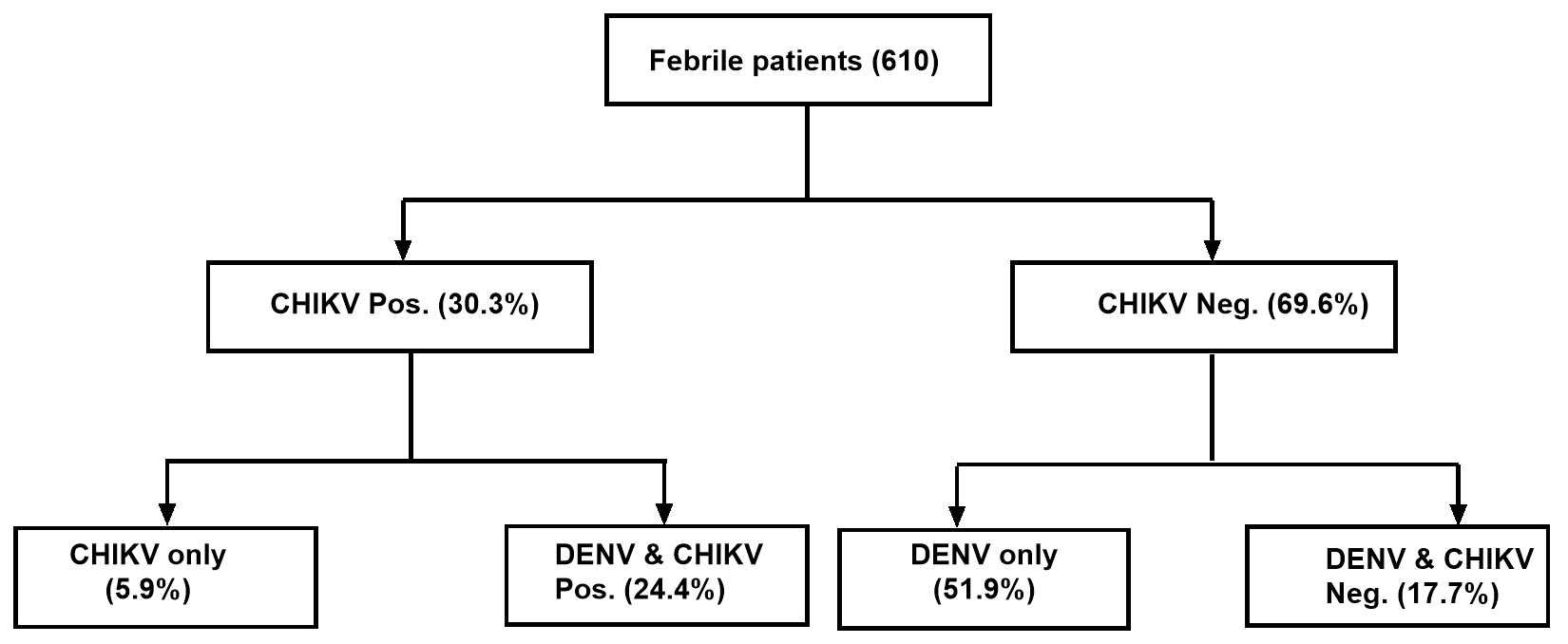

Supplement: S3 Fig — The flow chart illustrates the seroprevalence rates of DENV, CHIKV, and DENV–CHIKV infections. (TIF) [file pntd.0009961.s003.tif]

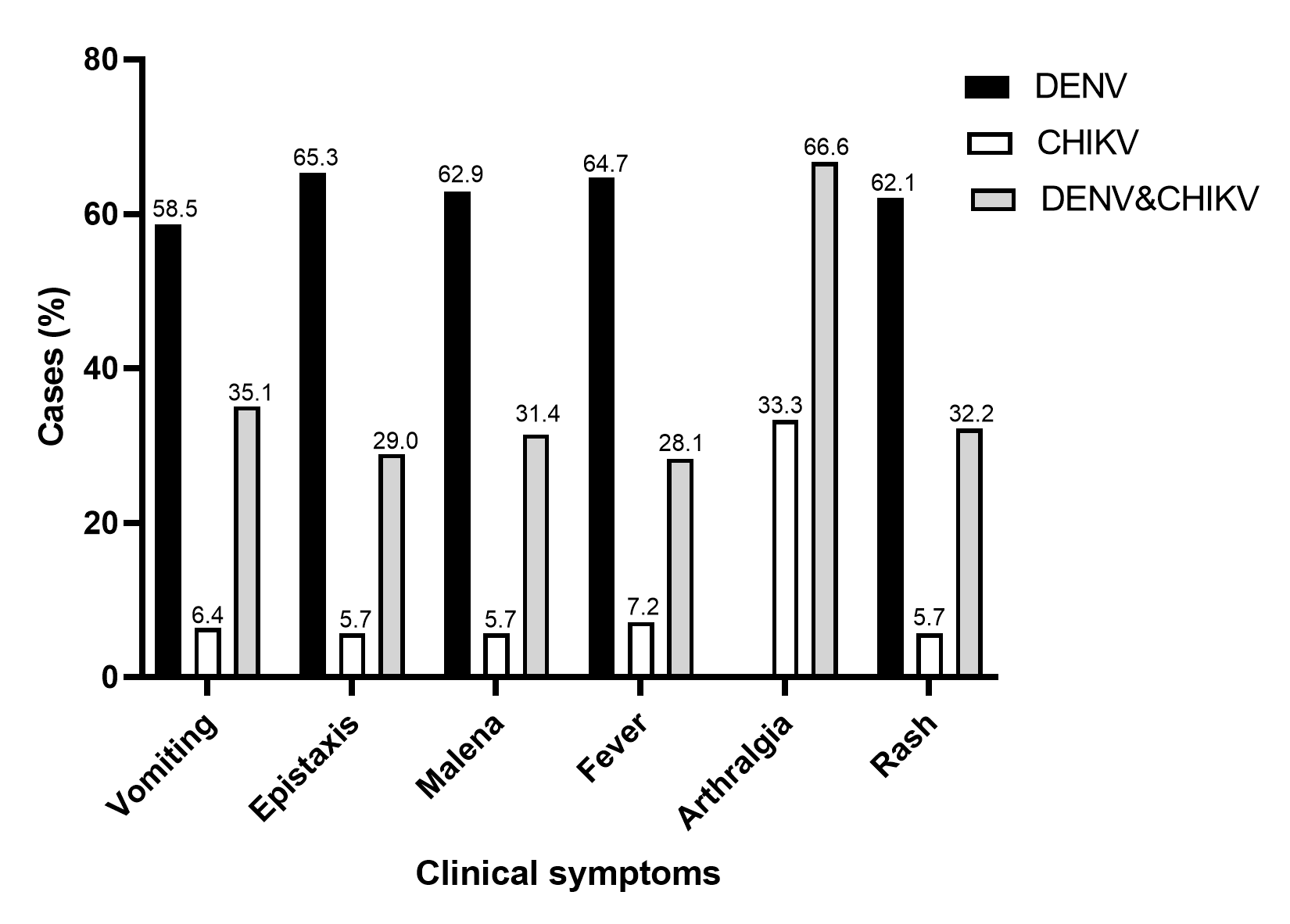

Supplement: S4 Fig — The prevalence rate of the clinical presentation among the febrile patients is indicated on each bar. (TIF) [file pntd.0009961.s004.tif]
