# Supplementary material for: Evidence of Chikungunya virus seroprevalence in Myanmar among dengue-suspected patients and healthy volunteers in 2013, 2015, and 2018
Source: PLoS Negl Trop Dis. 2021 Dec 1;15(12):e0009961. doi: 10.1371/journal.pntd.0009961 (PMC8635363; doi:10.1371/journal.pntd.0009961)
Supplement: S1 Table — The sensitivity of the in-house anti-CHIKV IgM capture ELISA was 98.3% (95% CI: 90.9%–100%) and specificity was 88.0% (95% CI: 71.8%–96.6%), with an accuracy of 94.6%. (DOCX) [file pntd.0009961.s005.docx]

|  |  | **Human anti-CHIKV Abcam IgM ELISA Kit (ab177848)** | | |
| --- | --- | --- | --- | --- |
|  |  | **Positive** | **Negative** | **Total** |
| **In-house anti-CHIKV IgM capture ELISA** | Positive | 58 | 4 | 62 |
|  | Negative | 1 | 29 | 30 |
| Total |  | 59 | 33 | 92 |
