# Supplementary material for: Evidence of Chikungunya virus seroprevalence in Myanmar among dengue-suspected patients and healthy volunteers in 2013, 2015, and 2018
Source: PLoS Negl Trop Dis. 2021 Dec 1;15(12):e0009961. doi: 10.1371/journal.pntd.0009961 (PMC8635363; doi:10.1371/journal.pntd.0009961)
Supplement: S2 Table — The sensitivity of the in-house anti-CHIKV IgG indirect ELISA was 94.2% (95% CI: 88.9%–97.5%) and specificity was 100% (97.8%–100%), with an accuracy of 97.4%. (DOCX) [file pntd.0009961.s006.docx]

|  |  | **FRNT_50_** | | |
| --- | --- | --- | --- | --- |
|  |  | **Positive** | **Negative** | **Total** |
| **In-house anti-CHIKV IgG capture ELISA** | Positive | 130 | 0 | 130 |
|  | Negative | 8 | 162 | 170 |
| Total |  | 138 | 162 | 300 |
