# Supplementary material for: Evidence of Chikungunya virus seroprevalence in Myanmar among dengue-suspected patients and healthy volunteers in 2013, 2015, and 2018
Source: PLoS Negl Trop Dis. 2021 Dec 1;15(12):e0009961. doi: 10.1371/journal.pntd.0009961 (PMC8635363; doi:10.1371/journal.pntd.0009961)
Supplement: S3 Table — Key: AIC, Akaike’s Information Criterion; BIC, Bayesian Information Criterion. The logistic regression model with four independent variables was selected because it had the lowest AIC and BIC values. The model was correctly classified at 66.4% and the goodness of fit test was p = 0.3773. (DOCX) [file pntd.0009961.s007.docx]

| **Model** | **AIC** | **BIC** |
| --- | --- | --- |
| **Age-group, Sex** | 1936.6 | 1952.6 |
| **Age-group, Sex, Site** | 1923.7 | 1945.1 |
| **Age-group, Sex, Site, Health status** | 1915.2 | 1941.8 |
| **Age-group, Sex, Site, Health status, Year** | 1916.8 | 1946.9 |
