# Supplementary material for: Evidence of Chikungunya virus seroprevalence in Myanmar among dengue-suspected patients and healthy volunteers in 2013, 2015, and 2018
Source: PLoS Negl Trop Dis. 2021 Dec 1;15(12):e0009961. doi: 10.1371/journal.pntd.0009961 (PMC8635363; doi:10.1371/journal.pntd.0009961)
Supplement: S4 Table — Key: AIC, Akaike’s Information Criterion; BIC, Bayesian Information Criterion. The logistic regression model with the four independent variables was selected because it had the lowest AIC and BIC values. The model was correctly classified at 68.5%, and the goodness of fit test was p = 0.2289. (DOCX) [file pntd.0009961.s008.docx]

| **Models** | **AIC** | **BIC** |
| --- | --- | --- |
| **Age-group, Sex** | 1890.3 | 1906.4 |
| **Age-group, Sex, Site** | 1878.6 | 1899.9 |
| **Age-group, Sex, Site, Health status** | 1867.3 | 1894.0 |
| **Age-group, Sex, Site, Health status, Year** | 1868.1 | 1899.1 |
