# Supplementary material for: Evidence of Chikungunya virus seroprevalence in Myanmar among dengue-suspected patients and healthy volunteers in 2013, 2015, and 2018
Source: PLoS Negl Trop Dis. 2021 Dec 1;15(12):e0009961. doi: 10.1371/journal.pntd.0009961 (PMC8635363; doi:10.1371/journal.pntd.0009961)
Supplement: S5 Table — The p-values highlighted in bold indicate significant values. (DOCX) [file pntd.0009961.s009.docx]

| **Variable** | **Crude Estimate (%)** | **95% CI** | | *p*-value | **Adjusted Estimate (%)** | **95% CI** | | *p*-value |
| --- | --- | --- | --- | --- | --- | --- | --- | --- |
| **Age (years)** |  | lower limit | upper limit |  |  | lower limit | upper limit |  |
| ≤5 | Ref |  |  |  | Ref |  |  |  |
| 6-15 | 0.05 | -0.008 | 0.11 | 0.09 | 0.11 | 0.04 | 0.17 | **0.002** |
| 16-45 | 0.20 | 0.14 | 0.27 | **0.0001** | 0.11 | -0.02 | 0.20 | 0.09 |
| ≥46 | 0.62 | 0.46 | 0.78 | **0.001** | 0.53 | 0.30 | 0.80 | **<0.0001** |
| **Region** |  |  |  |  |  |  |  |  |
| Mandalay | Ref. |  |  |  | Ref. |  |  |  |
| Myeik | 0.16 | 0.06 | 0.26 | **0.001** | 0.14 | 0.04 | 0.20 | **0.007** |
| Yangon | 0.22 | 0.17 | 0.27 | **0.0001** | 0.19 | 0.06 | 0.30 | **0.005** |
| **Gender** |  |  |  |  |  |  |  |  |
| Female | Ref. |  |  |  | Ref. |  |  |  |
| Male | 0.04 | -0.005 | 0.09 | 0.08 | 0.006 | -0.04 | 0.05 | 0.8 |
| **Health status** |  |  |  |  |  |  |  |  |
| Healthy  volunteers | Ref. |  |  |  | Ref. |  |  |  |
| Febrile  patients | -0.07 | -0.12 | -0.02 | **0.005** | 0.09 | 0.02 | 0.15 | **0.005** |
