# Supplementary material for: Evidence of Chikungunya virus seroprevalence in Myanmar among dengue-suspected patients and healthy volunteers in 2013, 2015, and 2018
Source: PLoS Negl Trop Dis. 2021 Dec 1;15(12):e0009961. doi: 10.1371/journal.pntd.0009961 (PMC8635363; doi:10.1371/journal.pntd.0009961)
Supplement: S7 Table — (DOCX) [file pntd.0009961.s011.docx]

| **Variable** | **Number** | **NAbs Titers** | |
| --- | --- | --- | --- |
|  |  | **Mean (95% CI)** | **GMT (95% CI)** |
| **Age (years)** |  |  |  |
| ≤5 | 268 | 137 (47-227) | 134 (86-209) |
| 6-15 | 701 | 408 (319-496) | 507 (391-658) |
| 16-45 | 554 | 850 (699-1003) | 953 (787-1152) |
| ≥46 | 21 | 1885 (498-3272) | 806 (352-1845) |
| **Region** |  |  |  |
| Mandalay | 927 | 336(268-409) | 384 (298-494) |
| Myeik | 104 | 465 (282-650) | 464 (270-797) |
| Yangon | 513 | 923 (756-1091) | 940 (779-1136) |
| **Gender** |  |  |  |
| Female | 602 | 492 (383-601) | 568 (435-741) |
| Male | 942 | 571 (473-668) | 609 (504-736) |
| **Health status** |  |  |  |
| Febrile patients | 610 | 390 (289-491) | 372 (282-491) |
| Healthy volunteers | 934 | 638 (536-739) | 761 (636-911) |
| Year |  |  |  |
| 2013 | 280 | 376 (234-516) | 389 (271-559) |
| 2015 | 330 | 402 (259-546) | 357 (234-545) |
| 2018 | 934 | 638 (536-739) | 761 (636-911) |
